# SYNCHRONIZED ACTIVITIES PROMOTE POSITIVE SENTIMENTS OF YOUNGER ADULTS TOWARDS OLDER ADULTS

**DOI:** 10.1093/geroni/igae098.3729

**Published:** 2024-12-31

**Authors:** Sarah Kim, Hsiao-Wen Liao

**Affiliations:** Georgia Institute of Technology, Atlanta, Georgia, United States; Georgia Institute of Technology, Atlanta, Georgia, United States

## Abstract

Studies have shown that younger adults often hold negative perceptions toward older adults, which can compromise intergenerational relationships and social harmony. The present study centers on interpersonal synchrony as a potential intervening venue. It adopted a pre- and post-test experimental design to test whether synchronous activities, specifically synchronous walking, promote positive sentiments of younger adults toward older adults. Components essential for forming positive interpersonal relationships regarding impression, perceived self-other closeness, connectedness, prosociality, and conversation engagement were examined. Participants (N = 51; 64.7% female) first completed a battery of questionnaires online assessing background information. After a few days, they were paired up with an older confederate and engaged in tasks. Participants were randomly assigned to one of the three conditions (i.e., synchronous walking, asynchronous walking, and no walking) and completed standard questionnaires assessing their sentiments toward the older confederate throughout experiments. A series of one-way repeated measures ANOVA, one-way ANOVA, and chi-square test of independence were conducted. Results revealed that synchronous walking significantly enhanced participants’ impressions, perceived self-other closeness, connectedness, and prosocial attitudes and behavior toward their older partner compared to asynchronous and no walking. The results remained significant when a set of individual difference factors was considered. This study extends past research on interpersonal synchrony to the context of aging. It sheds light on intervention practices for improving young people’s sentiment and behavior toward older adults, promoting intergenerational relationships.

